# Ultrafast dynamics and scattering of protic ionic liquids induced by XFEL pulses

**DOI:** 10.1107/S1600577521007657

**Published:** 2021-08-19

**Authors:** Kajwal Kumar Patra, Ibrahim Eliah Dawod, Andrew V. Martin, Tamar L. Greaves, Daniel Persson, Carl Caleman, Nicusor Timneanu

**Affiliations:** a Department of Physics and Astronomy, Uppsala University, Box 516, SE-751 20 Uppsala, Sweden; b European XFEL, Holzkoppel 4, DE-22869 Schenefeld, Germany; c School of Science, RMIT University, 124 La Trobe Street, Melbourne, VIC 3000, Australia; d Center for Free-Electron Laser Science, DESY, Notkestrasse 85, DE-22607 Hamburg, Germany

**Keywords:** radiation damage, molecular dynamics, non-local thermodynamic equilibrium, protic ionic liquids, X-ray free-electron lasers

## Abstract

X-rays are routinely used for structural studies through scattering, and femtosecond X-ray lasers can probe ultrafast dynamics. We aim to capture the femtosecond dynamics of liquid samples using simulations and deconstruct the interplay of ionization and atomic motion within the X-ray laser pulse. This deconstruction is resolution dependent, as ionization influences the low momentum transfers through changes in scattering form factors, while atomic motion has a greater effect at high momentum transfers through loss of coherence. Our methodology uses a combination of classical molecular dynamics and plasma simulation on a protic ionic liquid to quantify the contributions to the scattering signal and how these evolve with time during the X-ray laser pulse. Our method is relevant for studies of organic liquids, biomolecules in solution or any low-*Z* materials at liquid densities that quickly turn into a plasma while probed with X-rays.

## Abstract 2

This radiation damage study uses a hybrid approach combining molecular dynamics with non-thermal plasma simulations to follow the femtosecond dynamics and X-ray scattering from a protic ionic liquid as it is investigated by an intense XFEL pulse.

## 1. Introduction   

The idea of using X-ray free-electron lasers (XFELs) for the determination of protein structures was introduced at the turn of the century (Neutze *et al.*, 2000 ▸). With the glorious goal to perform high-resolution imaging of single protein molecules, considerable effort has been made to develop and build XFEL facilities that today are available to the scientific community. Even though high-resolution single-particle imaging using XFELs has not been achieved yet, XFEL sources have created numerous exciting new research directions. The most prominent that should be mentioned is serial femtosecond crystallography, SFX (Chapman *et al.*, 2011 ▸; Boutet *et al.*, 2012 ▸), which uses the high photon numbers and the short pulses of XFELs to solve protein structures using nanometre-sized crystals using an approach similar to conventional crystallography. The XFEL pulses can have a peak brilliance that is several orders of magnitude higher than that at fourth-generation synchrotron facilities. This feature allows for investigations of matter under extreme conditions, as any sample put in the beam will be highly ionized and heated while the X-ray pulse is still propagating through it (Beyerlein *et al.*, 2018 ▸). Since biomolecules are sensitive to radiation damage, the high ionization due to the intense X-ray pulse leads to complications in the reconstruction process, thus limiting the achievable resolutions (Howells *et al.*, 2009 ▸; de la Mora *et al.*, 2020 ▸; Östlin *et al.*, 2019 ▸).

For crystalline samples the damage does not affect the structural resolution as much as was initially thought (Neutze *et al.*, 2000 ▸), due to the self-terminating effect (Barty *et al.*, 2012 ▸). Since crystallography uses the photons diffracted into the Bragg spots, it will only record a structurally coherent sample. As soon as the crystalline structure is lost, it will no longer Bragg-diffract. This phenomenon makes the sample somewhat robust to global damage (Barty *et al.*, 2012 ▸; Caleman *et al.*, 2015 ▸; Chapman *et al.*, 2014 ▸). However, if the damage manifests itself in a similar way in multiple unit cells of the protein crystal, this might be recorded in the Bragg diffraction. This has been seen in hot spots in the crystal, where atoms with higher ionization cross sections are situated (Nass *et al.*, 2020 ▸, 2015 ▸; Galli *et al.*, 2015 ▸). This so-called specific damage was seen experimentally using time-resolved measurements, and it indicates that the sulfur–sulfur distance in a lysozyme sulfur bridge is elongated by around 1 Å in 60 fs. These measurements also agree well with several theoretical descriptions (Nass *et al.*, 2020 ▸; Caleman *et al.*, 2020 ▸).

Self-terminating Bragg diffraction will not happen if the sample is non-crystalline. For single-particle imaging using XFELs, theoretical calculations have suggested that a similar gating effect applies to single-molecule diffraction with respect to spatially uncorrelated damage processes like ionization and ion diffusion (Martin *et al.*, 2015 ▸). This begs the question: what is the situation for liquids? An entirely different category of structural investigations with XFELs involves liquid samples, or samples suspended in a liquid carrier or buffer. The first study that followed the structural changes in liquids due to XFEL pulses was made on water (Beyerlein *et al.*, 2018 ▸), where the sample turned into a plasma, undergoing an ultrafast phase transition at liquid density. In this study we consider the dynamics of a liquid sample that contains organic ions. X-ray scattering from this type of sample gives a strong signal compared with single-particle imaging, as it involves a large number of organic ions in the interaction volume; however, these would be typically disordered and it is not expected to be subject to any gating effect. The ultrafast damage processes of ionization due to the XFEL and the fast ion diffusion is expected to average out in the liquid, thus the overall scattering signal will only carry reduced information about the detailed dynamics.

In this paper we use computational tools to unravel the detailed dynamics in an organic liquid, and disentangle the contribution to the overall scattering signal coming from the different damage processes that happen in the liquid. Our approach is similar to the hybrid model we employed to describe the nonthermal heating of water which we compared with the experimental results (Beyerlein *et al.*, 2018 ▸). There are several methods that describe ultrafast dynamics at high intensities, using molecular dynamics/plasma hybrid approaches (Hau-Riege *et al.*, 2012 ▸; Nass *et al.*, 2020 ▸; Kozlov *et al.*, 2020 ▸), or using self-consistent field simulations of atomic and molecular orbitals (Hao *et al.*, 2015 ▸; Grånäs *et al.*, 2019 ▸). The hybrid methods have the advantage of being scalable to large systems at a low computational cost, while the latter have the advantage of providing detailed chemical dynamics but are computationally expensive and are suitable for applications to small systems.

The choice of sample is a protic ionic liquid, motivated by an increasing interest in their use as solvents for biomolecules, such as proteins (Han *et al.*, 2021 ▸), and due to the interesting liquid nanostructure which is present for many protic ionic liquids (Greaves *et al.*, 2010 ▸). The second motivation is to develop a methodology for investigating samples in liquid phase, by combining two computational tools with complementary strengths: molecular dynamics simulations to follow femtosecond atomic structural changes and plasma simulations to follow the ionization and electronic changes in the sample. These are described in detail in the *Methods* section below[Sec sec2], and their application on a particular protic ionic liquid investigated by XFEL pulses with experimental parameters is presented in the *Results* section[Sec sec3].

## 2. Methods   

**Figure 1:**
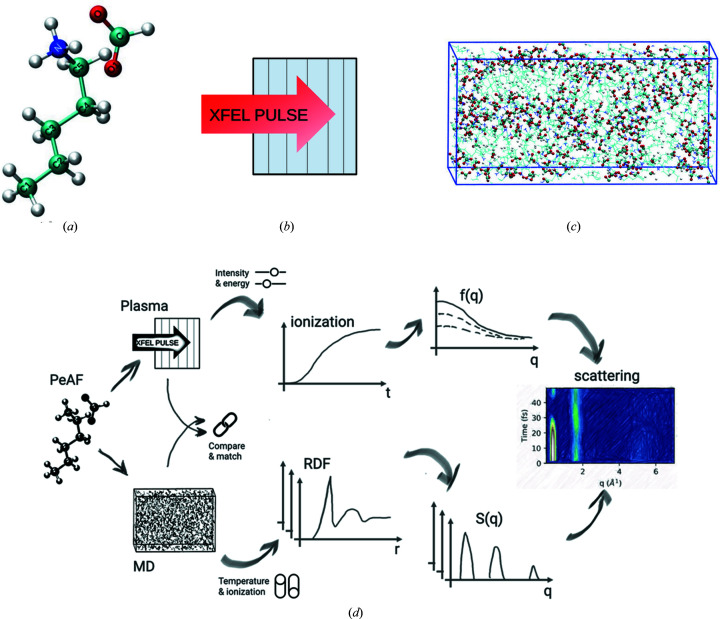
(*a*) A snapshot of pentylammonium formate (PeAF) consisting of a pentylammonium cation and a formate anion. The molecular ions are shown in stick representation with the following colour code: cyan, blue, red and white for carbon, nitrogen, oxygen and hydrogen, respectively. (*b*) A schematic representation of 1D simulation geometry of plasma simulation where the homogeneous sample is divided into six distinct zones separated by black thin lines. The zones contain only information on the stoichiometry of the sample (H:15, C: 6; N:1, O:2) and no molecular structure. (*c*) Snapshot of a classical MD simulation box containing 300 PeAF ion pairs settled arbitrarily after initial minimization and equilibration. Colour coding as in (*a*). (*d*) Sketch of the simulation and analysis flow, combining the plasma and MD simulations, to obtain the final scattering from the liquid. X-ray beam intensity and energy are represented as ‘sliders’ and were varied to obtain the ionization dynamics and scattering form factors. Temperature and ionization in MD are represented as ‘switches’ and show different simulation scenarios for calculating the RDFs and structure factors.

We present a multistep model which links different methodologies. An overview of the system, simulations and workflow is presented in Fig. 1 ▸. At step (1), we start with plasma simulation of the sample. This is done using the radiation transfer code *CRETIN* (Scott & Mayle, 1994 ▸; Scott, 2001 ▸) that models a highly energetic ultrafast XFEL radiation pulse onto the sample. The output of the simulation includes the electronic states of the elements, and the ion and electron temperatures during the XFEL radiation. At step (2), we gather all the information from the previous step, particularly the ionization states for each element and compute the atomic and ionic form factors. The first two steps are sketched in the top branch of Fig. 1 ▸(*d*), which are repeated for several X-ray beam intensities and X-ray photon energies. At step (3), we perform classical molecular dynamics (MD) simulation of the system using the *GROMACS* package (van der Spoel *et al.*, 2005 ▸; Hess *et al.*, 2008 ▸). This allows us to follow the time evolution of the positions of the atoms. Using the output from the MD simulations, in step (4), we calculate the radial distribution functions (RDFs) and corresponding structure factors. These two steps are shown in the lower branch of Fig. 1 ▸(*d*), and have been repeated for several X-ray beam parameters, several system setups, and a high number of iterations to increase the statistics. To take advantage of the advanced physical description of the ionization processes in the plasma code, we use the ionization rates from the plasma simulations in step (1), and match the ionization in our MD simulation to those. Finally, at step (5), we combine the form factors from step (2) and RDFs from step (4), and use those to calculate the scattering in terms of time-dependent structure factors to obtain the final scattering signals.

### 2.1. Plasma simulations   

To mimic the interaction between the high-intensity X-rays and the sample we have used a non-local thermodynamic equilibrium (non-LTE) radiation transfer code called *CRETIN* (Scott & Mayle, 1994 ▸; Scott, 2001 ▸), commonly referred to as a plasma code. This code has been successfully compared with experimental measurements of the interaction between XFEL radiation and matter (Barty *et al.*, 2012 ▸; Caleman *et al.*, 2015 ▸; Beyerlein *et al.*, 2018 ▸). *CRETIN* has been benchmarked against several other similar codes (Larsen & Lane, 1994 ▸; Chung *et al.*, 2005 ▸; Gilleron & Piron, 2015 ▸). The simulation maps the footprints of ionization, electronic level populations, radiation spectra, opacities, radiation transports and heating rates (Caleman *et al.*, 2011*a* ▸,*b* ▸). The code uses a screened hydrogenic model for the element composition of the sample. It simulates the Auger decay process by taking lifetimes of hollow atoms into account. The model accounts for changes in the absorption cross section due to electron excitation and depletion of electron states as well as continuum lowering. It also includes processes like secondary ionization, for example through electron–ion collision. Instant thermalization of electrons is assumed, and the electrons are assumed to follow a Maxwellian energy distribution. The time evolution of atom and ion energies in the system are affected by the choice of coupling coefficient. In this model, the coefficient is calculated with Spitzer’s formula (Spitzer, 1956 ▸). The model further contains a description of the lowering of continuum edges calculated by the Stewart–Pyatt formula (Stewart & Pyatt Jr, 1966 ▸), which is a common approximation that has been accepted for both experiments and more detailed models (Nantel *et al.*, 1998 ▸).

The model considers the average atomic composition of the sample and does not require structural information for the system. It does not treat the atomic bond breaking process in the sample. The code is developed to work in the plasma energy range, which is higher than the average binding energy of a molecule. Also, one of the benefits of using such a plasma approach is that it can treat large biomolecular systems like proteins and enzymes at an affordable computational cost (Lomb *et al.*, 2011 ▸). However, to get an idea of the local structural changes and to study the collective motion of the system we combine the output of our plasma code with classical MD simulations. Several hybrid approaches have been taken for XFEL MD and have been presented earlier (Hau-Riege *et al.*, 2012 ▸; Nass *et al.*, 2020 ▸; Kozlov *et al.*, 2020 ▸). The current study builds on and expands our previous study (Beyerlein *et al.*, 2018 ▸), and offers the advantage of using state-of-art MD potentials, constraints and equilibration schemes from *GROMACS*, in combination with the non-local thermodynamic equilibrium plasma code.

In this study, one-dimensional plasma simulation of pentylammonium formate (PeAF) was carried out to follow how the ionization due to XFEL and the dynamics of the system affects the scattering signal. The simulation geometry was divided into six simulation zones where each zone has the same atomic composition corresponding to PeAF [C_6_H_15_N_1_O_2_ see Fig. 1 ▸(*a*)] with density 0.95 g cm^−3^ at room temperature (Greaves *et al.*, 2006 ▸). A qualitative picture of such a one-dimensional plasma simulation geometry is shown in Fig. 1 ▸(*b*). Each zone contains only the information about the stroichiometry of PeAF and distinctly behaves as a continuum, with charge and mass being conserved but heat and radiation able to transfer between the zones. Simulation input parameters can be found in examples of generator files for *GROMACS* and *CRETIN* in the supporting information, where both the sample parameters (relative atomic composition, density, geometry) as well as the XFEL parameters are given. Another example of a generator file for *CRETIN* can be seen in our previous study on water (Beyerlein *et al.*, 2018 ▸).

The model allows the zones to expand during the simulation. The sample thickness for this study was taken as 5 µm, which is a comparable size to the diameter of the jet in XFEL experimental set-ups (Liang *et al.*, 2015 ▸; DePonte *et al.*, 2008 ▸; Boutet & Williams, 2010 ▸). The sample was exposed to a 50 fs-long pulse with photon energy ranging between 5 keV and 9.5 keV and intensity ranging from 1 × 10^17^ to 1 × 10^20^ W cm^−2^. The choice of such XFEL parameters is relevant both for the currently operating Linac Coherent Light Source (LCLS) and European XFEL (Liang *et al.*, 2015 ▸; Schneidmiller & Yurkov, 2011 ▸). The lower-intensity end of the simulation corresponds to the intensity of the CXI endstation where many experiments have been performed to date (Redecke *et al.*, 2013 ▸; Boutet *et al.*, 2012 ▸; Kern *et al.*, 2015 ▸), while the higher end of the simulation corresponds to intensities in the 100 nm focus (Nass *et al.*, 2016 ▸). There are several XFELs with a peak brilliance slightly lower than European XFEL [1 × 10^33^ versus 5 × 10^33^ photons s^−1^ mm^−2^ mrad^−2^ (0.1% bandwidth)^−1^], such as SACLA, SwissFEL, PAL-XFEL and SHINE^1^, where our current work could still be relevant provided the experimental endstations have a high X-ray transmission and a sub-micrometre focus.

### 2.2. Form factors   

X-rays are scattered by the electron cloud of an atom and the scattering amplitude depends on the atomic number (*Z*) of the atoms in a sample, where atoms with more electrons (higher *Z* values) scatter more. Atomic form factors are defined as the Fourier transform of the electron density of an atom, 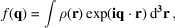
where ρ(**r**) is the electron charge density and **q** is the momentum transfer. If the atoms of a sample are assumed to be spherically symmetric and isolated, the general expression for the atomic form factor is 
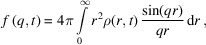
where ρ(*r*, *t*) is the radial and time-dependent charge distribution. The atomic form factor can be approximated using the Cromer and Mann method (Cromer & Mann, 1968 ▸), which is based on the fitting of experimental data with a sum of Gaussians. For each ionized state, they are described as 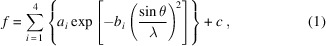
where 2θ is the scattering scattering angle, λ is the wavelength of the incident radiation and *a*_*i*_, *b*_*i*_ and *c* are Gaussian parameters. To get the full form factor of an element, all the ionized states are summed up as (Caleman *et al.*, 2015 ▸) 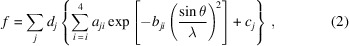
where *d*_*j*_ is the fraction of atoms and ions in each ionization state *j*, and carries time dependence.

### 2.3. Molecular dynamics simulations   

We have employed classical MD simulations to study the structural dynamics of the sample. In classical molecular dynamics, a molecular geometry is defined and a force field is used to calculate the interactions between the constituents, which is thereafter used to propagate the positions of the atoms numerically. We used the Molefacture module in *VMD* (Humphrey *et al.*, 1996 ▸; Stone, 1998 ▸) to create the PeAF structure. Once the desired PeAF structure was created, the next point was to create the starting structure and its corresponding topology. The online server ‘ACPYPE’ was used to create the initial structure and topology files (Da Silva & Vranken, 2012 ▸). It is designed to develop topology parameters for any organic chemical compound and is able to work for different MD platforms like *GROMACS* (van der Spoel *et al.*, 2005 ▸), *CHARMM* (Brooks *et al.*, 2009 ▸) and *AMBER* (Case *et al.*, 2005 ▸). The versatile usage and the scope of generating topology parameters for molecules by ACPYPE has created a lot of interest for studying relatively new/lesser-known compounds via MD simulations. For our studies, we created a generalized amber force field (GAFF) which provides parameters suitable for small molecules that are compatible for *GROMACS* simulations. Recent studies indicate that GAFF can predict different properties of several ionic liquids, which suits our sample (Sprenger *et al.*, 2015 ▸; Reddy & Mallik, 2020 ▸; Liu *et al.*, 2020 ▸; Luo *et al.*, 2020 ▸).

The simulations are performed with the *GROMACS* software package (van der Spoel *et al.*, 2005 ▸; Hess *et al.*, 2008 ▸). The initial size of the simulation box was 10 nm × 10 nm × 10 nm with a total of 300 PeAF cation and anion pairs. First an energy minimization of the system was performed using the steepest descent. Then, both *NVT* [the amount of substance (*N*), volume (*V*) and temperature (*T*) are conserved also known as canonical ensemble] and *NPT* [the amount of substance (*N*), pressure (*P*) and temperature (*T*) are conserved also known as isothermal-isobaric ensemble] equilibrations are done for more than 10 ns before the production run. Periodic boundary conditions were applied throughout the simulation along with the LINCS algorithm which constrains the hydrogen bonding (Hess *et al.*, 1997 ▸). The particle mesh Ewald (PME) algorithm (Darden *et al.*, 1993 ▸; York *et al.*, 1993 ▸) was applied for long-range electrostatics. For temperature coupling we have applied the modified Berendsen thermostat with a time constant τ = 0.3 ps and reference temperature 298 K (Berendsen *et al.*, 1984 ▸; Berendsen, 1991 ▸). Similarly, for pressure coupling in *NPT*, the Nose–Hoover algorithm was applied with a time constant τ = 0.2 ps and a reference pressure at 1 bar (Nosé & Klein, 1983 ▸).

To mimic the dynamics in the liquid caused by the ionization from the XFEL radiation, we used a version of *GROMACS* that included an ionization algorithm, described by Neutze *et al.* (2004 ▸), based on *GROMACS 3.3.3* (Lindahl *et al.*, 2001 ▸). This approach uses equilibrium parameters for the force field for both the equilibrium (regular MD) and non-equilibrium simulations with the ionization algorithm. In the latter, the change due to the X-ray pulse is the rapid increase of the Coulomb interaction since the atoms are ionized. We assume here that the Coulomb interaction will dominate the dynamics of the system, when the system is highly ionized. The same approach was applied in earlier studies (Neutze *et al.*, 2000 ▸; Bergh *et al.*, 2004 ▸; Caleman *et al.*, 2011*a* ▸,*b* ▸; Östlin *et al.*, 2019 ▸), with the exception that here we compared and matched the ionization rates with the plasma simulations. This way we can take advantage of the well developed description of the photon–matter interaction that the non-LTE code provides, and combine that with the MD package from *GROMACS* to follow the dynamics in terms of atomic positions as a function of time.

### 2.4. RDF and static structure factors *S*(*q*)   

The RDF is a tool to study the structure of a liquid. Given the coordinates of the atoms one can compute the RDFs for different pairs of elements present in a sample. In simple terms, the RDF describes how the density varies as a function of distance from a reference particle or a group of particles. In general the idea is to find out how many particles are present within the distance *r* and *r* + d*r* away from the reference particle. For a homogeneous and isotropic system, the RDF depends only on the distance between particles (Sellberg, 2014 ▸), 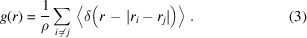
The scattering of incident radiation caused by a material can be described mathematically as the static structure factor or simply structure factor. It is directly related to the RDF via Fourier transform, the Fourier density correlation 
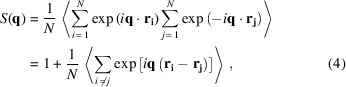
where *N* is the number of particles and **q** is the moment transfer. We can directly calculate *S*(*q*) from the RDF as 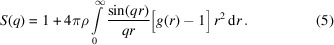



### 2.5. Scattering and time-dependent structure factors   

In coherent scattering with XFELs, the scattered intensity (*I*) is mainly affected by ionization and atomic displacement. While ionization changes the form factor *f*, atomic displacement alters the structure factor *S* of the sample. In a liquid the molecules are randomly oriented, and the intensity is averaged over all possible orientations. According to the theory of scattered intensity (Debye, 1915 ▸), the scattered intensity from the interaction is proportional to the form and structure factor as 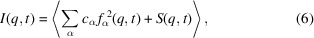
where α represents number of different elements of the sample, with *c*_α_ being the number density of element α. The total structure factor *S*(*q*, *t*) is a function of momentum transfer and time which accounts for all the available elements of the sample. When normalized by the atomic scattering part of equation (6), it can be defined as (Wikfeldt, 2011 ▸; Sellberg, 2014 ▸) 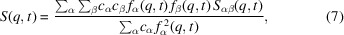
with *S*_αβ_ being the element-wise contribution to the total structure factor from elements α, β and can be calculated as the Fourier transform of time-dependent RDF *g*_αβ_, 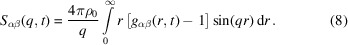
Figure 1(*d*) shows how the time-dependent RDFs *g*_αβ_ are extracted directly from MD simulations, for both the static and dynamic cases that we consider. For better statistics for individual time steps, the RDFs can be computed from many parallel trajectories. The figure also depicts how the scattering form factors *f*_α_(*q*, *t*) and their relative weights are calculated following the plasma simulations.

## 3. Results   

Our study employs molecular dynamics and plasma physics with different purposes. Plasma simulations were performed on a PeAF system to measure the electron and ion populations, the corresponding electron and ion temperatures, and ionization states of each elements, which further led to calculations of the atomic and ionic form factor. In parallel, MD was employed to calculate the local atomic motion of the sample. The idea is to calculate the RDF of the atomic groups which in turn leads to the initial structure factor of the system. Finally, we incorporate both the results from MD and plasma simulations to obtain a time-dependent structure factor and study scattering intensity from the sample as a function of time. When modelling the MD systems, we created several parallel simulation conditions starting from simpler to more advanced within the limitations of our hydrid approach. The idea behind building different systems is to deconstruct the interactions of the scattering mechanism and to learn how each of those influences the scattering process. The details of these simulation conditions are given in Table 1 ▸, and Fig. 1 ▸(*d*) shows schematically how these systems are obtained with the ‘switches’ for temperature and ionization. This results section gradually showcases the results of the basic system A-cold up to the more complex system D-hot. The system A-cold comprises MD simulations at room temperature, which give a static RDF and the ionization is taken separately from the plasma simulation, while the system D-hot includes high-temperature MD simulations, which give a dynamic RDF with atomic displacement calculated from similar ionizing environment as plasma, and with ionization form factors from plasma simulations. The intermediate systems B and C were used as checking points in our simulations; however, we will not show any results here. In the following we will present the results from the A-cold system, that can be compared with experiments at synchrotrons, and the D-hot system that can be compared with experiments at XFELs.

**Figure 2:**
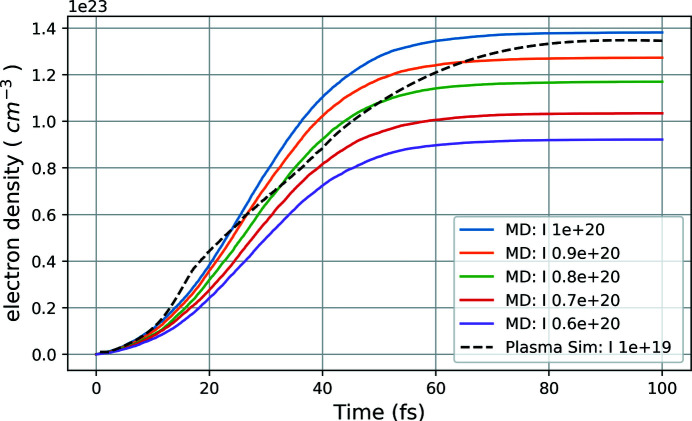
Comparison of electron density versus time plot between plasma simulation and MD simulation. The black dashed line represents the plasma simulation results at pulse intensity 1 × 10^19^ W cm^−2^, while the solid lines represent the MD simulations at different pulse intensities (nominally expressed in W cm^−2^).

**Figure 3:**
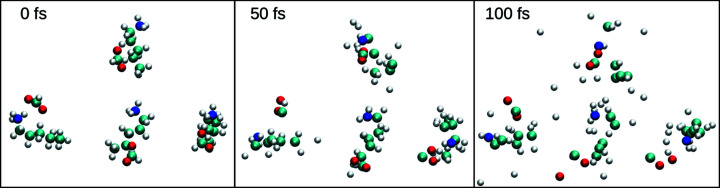
Snapshots of different time frames from MD simulations following XFEL ionization. Each frame shows a close-up view of four neighbouring PeAF cation and anion pairs where the atoms are represented by colour-coded spheres: carbon in cyan, hydrogen in white, oxygen in red and nitrogen in blue. It indicates how the system quickly turns into a plasma and explodes as a function of time due to the XFEL pulse.

Figure 2 ▸ describes the comparison between the electron density in the system versus time from both the MD and plasma results. The plot indicates that the electron density from plasma simulation (the black dashed line) with pulse intensity 1 × 10^19^ W cm^−2^ closely matches with the electron density from MD simulation (solid blue line) with a nominal pulse intensity 1 × 10^20^ W cm^−2^ which corresponds to almost a one order higher intensity with respect to the plasma calculations. This discrepancy can be understood from the current limitation of the MD simulations which could only ionize through the process of photoionization and Auger decay, while in the plasma case all the primary and secondary effects of photoionization, Auger processes, secondary ionization and electron cascades are included as well. Thus with almost one order higher nominal intensity, the MD ionizaton matches closely with the more realistic plasma simulations. The idea is to compare both the ionization results to match the pulse parameters for the MD simulation to achieve a similar ionizing environment as in plasma. Once these were known, we performed more than 100 independent MD simulations with those pulse parameters and calculated the time-dependent RDFs for analysis of the system D-hot. The 100 independent MD setups were created for each set of particular pulse parameters (photon energy, intensity, pulse duration), had the same initial condition from equilibrium and were run by generating initial random seeds for stochastic ionization for each simulation. Figure 3 ▸ shows snapshots of four neighbouring PeAF ion pairs inside the MD simulation box at different time frames of the XFEL-induced explosion simulation. Each molecule is represented by a colour code with atoms as spheres. With a pulse duration of 50 fs, we see the molecules boiling up and the atoms start diffusing from their initial structure as a function of time.

### 3.1. Radial distribution and structure   

For a deeper understanding of the structure and how this becomes encoded in scattering, we first look at the classical molecular dynamics. In a preliminary step, we run two independent simulation boxes with different sizes to check the validity of our statistics and optimize the computational time: a small box with a 7.3 nm × 2 nm × 3.5 nm volume accommodating 300 PeAF ion pairs (cations and anions) with a total of 7200 atoms, and a larger box having size 6.8 nm × 6.9 nm × 6.7 nm, containing 1200 molecules with a total of 28800 atoms. A comparison of the results for the two box sizes for system A-cold shows that the observables of interest are nearly identical within our required statistical accuracy. For computational efficiency, we have used the simulations on the smaller box to include all the four different model systems as described in Table 1 ▸. The structural difference of these four systems are then studied by calculating the RDF of different pairs of elements present. Particularly for the system D-hot, the time-dependent RDFs were calculated as averages of the 100 independent MD simulations. At any particular time, all the corresponding frames from the independent simulations were grouped together and used to calculate the RDF at that particular time step.

**Figure 4:**
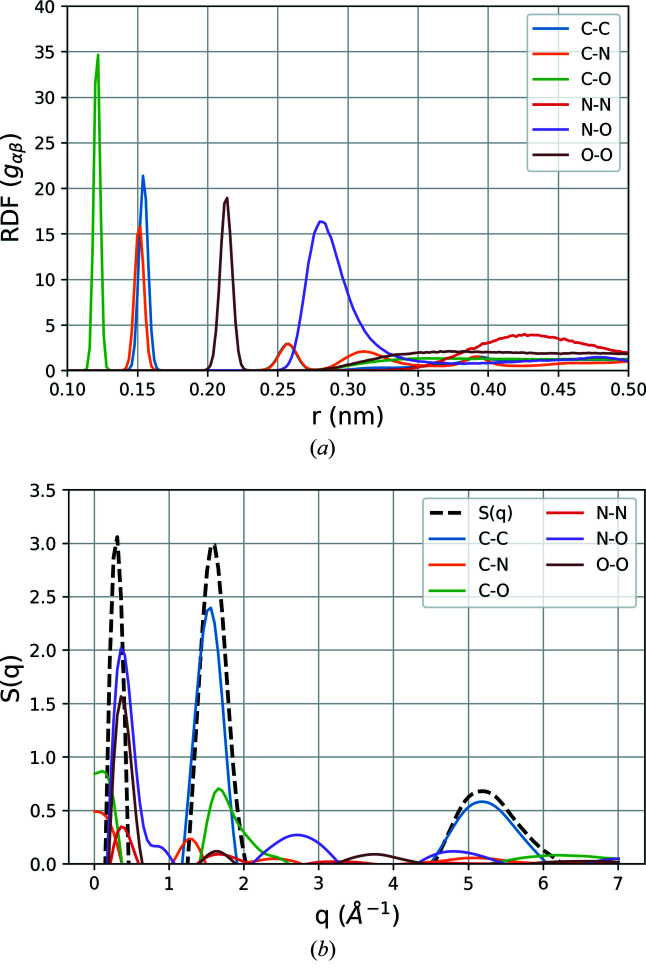
(*a*) Static radial distribution function (RDF) of element pairs present in PeAF, calculated from a classical MD simulation trajectory of the A-cold system. (*b*) Corresponding structure factor contributions from the element pairs calculated as the Fourier transform of the RDFs. The black dashed line represents the total static structure factor *S*(*q*) for the A-cold system with three predominant features, at lower *q* (∼0.5 Å^−1^), intermediate (∼1.5 Å^−1^) and at higher *q* (∼5 Å^−1^).

Figure 4 ▸ shows the RDF plots for different elements and corresponding static structure factor contributions to the system A-cold. The RDF plot shows that the system is well equilibrated and all the atom–atom distances are well developed having very sharp peaks. In particular, the width of the C–C, C–O, O–O distance distribution indicates that the distance between the corresponding atoms are consistent throughout the simulation, depicting the atomic arrangement to be stable in terms of the dynamics of the system. However, the distance between N–N bonding seems to be relatively widely distributed indicating that the interatomic N–N distances between different molecules are mainly affected by the thermal vibration. The secondary and tertiary peaks of the C–N distance represent the distances between each of the further carbon atoms present in the pentylammonium chain to the N atom at the end of the chain.

Figure 4 ▸(*b*) shows all the contributions to structure factors from the corresponding *g*_αβ_, calculated using equation (8). From the different contributions of the atomic pairs, we identify the main features in the final signal, which follows closely the features observed experimentally with X-ray scattering at synchrotrons (Greaves *et al.*, 2006 ▸). The first peak at low scattering vector *q* = 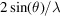 at approximately 0.5 Å^−1^ is associated with the ordering in the ionic liquid, and comes mainly from the N–O, O–O and N–O long-range correlations. The following peak (1.5–2 Å^−1^) is associated mainly with the structure of the cations and anions, and consists of diffraction from C–C and C–O, and the third peak at 4.5–5 Å^−1^ has contributions from C–O and N–O, respectively. There is a also a small contribution at around 2.5 Å^−1^ from the N–O pair.

### 3.2. Effects of ionization   

To investigate the ionization in the sample and the effects on the scattering intensity due to the XFEL beam, we turn to the plasma simulations. The non-local thermodynamic equilibrium atomic kinetics and radiation transfer code performed in 1D assumes that a plasma is formed within the first few femtoseconds. We have made a systematic investigation of different XFEL parameters like the photon energy (5 to 9.5 keV), ultrashort pulses (50 fs) and radiation intensity (1 × 10^17^ to 1 × 10^20^ W cm^−2^), with the diameter of the spot size being assumed to be 1 µm.

**Figure 5:**
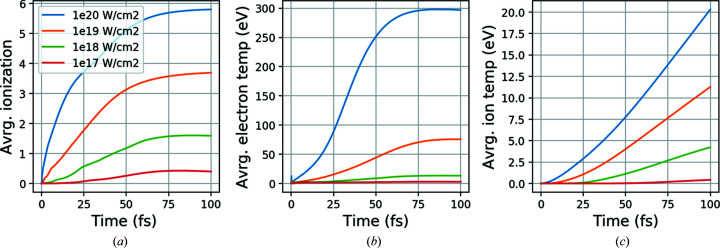
(*a*) Average carbon ionization, (*b*) average electron temperature and (*c*) ion temperature as a function of time from the plasma simulation at 5 keV photon energy with different pulse intensities.

In Fig. 5 ▸(*a*), we have plotted the average ionization of carbon in the sample at 5 keV photon energy with different XFEL intensities spanning several orders of magnitude. The evolution of the ionization for all other elements shows a similar pattern, where the probability of ionization increases with increasing atomic number. In Figs. 5 ▸(*b*) and 5(*c*), the average electron and ion temperature of the sample are shown for the same XFEL intensities. As expected, we find a clear and substantial increase of the electron temperature for given energy and intensities. The electron temperature reaches the highest values for lowest energy (5 keV, where the absorption is the highest) and highest intensity (1 × 10^20^ W cm^−2^) and shows almost an exponential growth particularly with a difference of order two compared with at lower intensities. The ratio of photoabsorption to coherent scattering also increases for lower photon energy leading to more energy deposited in the sample. In addition, a high-intensity pulse always has a direct correlation with a high sample temperature. The considerable temperature difference between ion and electron in the plot indicates the large mass difference between them, and they will reach thermal equilibrium on picosecond time scales.

### 3.3. Time-dependent form factors.   

During the XFEL pulse, the fraction of atoms and ions in different ionization states for different elements of the sample are extracted from the plasma simulations and are used to calculate the form factors as a function of time and momentum transfer. As hydrogen has an extremely low interaction cross section, it does not contribute significantly to the scattering process and we have neglected it in our calculations.

**Figure 6:**
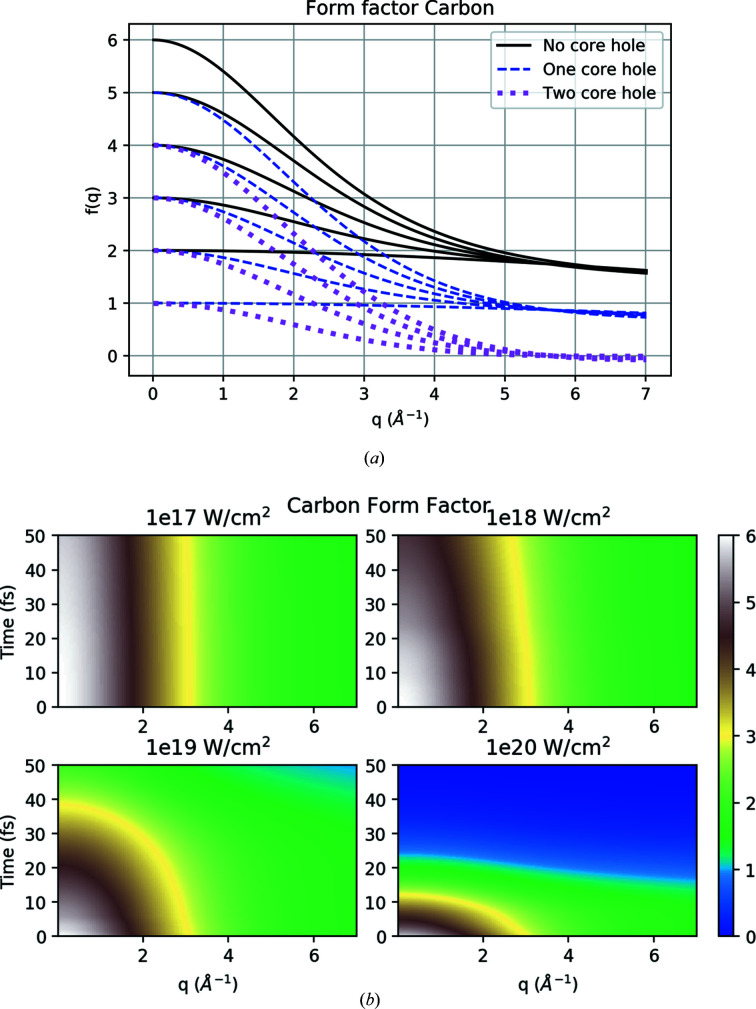
(*a*) Cromer and Mann form factors for different ionization states of carbon as a function of resolution *q*. Solid black lines represent states with no core hole in the *S* orbital, dashed blue with one core hole and dotted purple represent states with two core holes. The value at *q* = 0 represents the number of bound electrons for each of the states. (*b*) Time dependence of carbon form factors following plasma simulations at 5 keV photon energy and for different XFEL intensities.

Figure 6 ▸(*a*) shows the form factors of C for different ionization states as described by the Cromer and Mann method (Cromer & Mann, 1968 ▸) in equation (1). We have obtained similar form factors for all the other elements. The plot indicates that for higher *q* values the form factors are mainly dependent on how many electrons occupy the 1*s* orbital, and at low *q* values the net charge of the atom plays a significant role.

Figure 6 ▸(*b*) depicts the time evolution of the carbon form factors from plasma simulations with 5 keV photon energy and different pulse intensities, where the intensity of the XFEL beam is seen to have a significant influence on the evolution. For the lowest intensity 1 × 10^17^ W cm^−2^, the sample is not ionized severely, leading to an almost unperturbed form factor for lower *q* values. These results follow the ionization from Fig. 5 ▸(*a*) well, where, at low intensity, carbon was barely ionized. As the intensity increases, we see a quick decrease in the form factor due to the ionization process at low *q* values. At higher *q* values, we see the same trend but with a lesser rate of decrease compared with the lower *q* values. For the highest intensity (1 × 10^20^ W cm^−2^), the form factor for all the *q* values soon becomes zero half way through the pulse. We also find the form factors for different photon energies, and intensities decrease in a similar way, but at different rates. The change is slower for an increase in photon energy compared with the increase in intensities. Further, the rate of change of the form factors for different elements depends on the atomic number (*Z*); for example, oxygen has a faster rate of ionization compared with nitrogen and carbon.

### 3.4. Dynamics of structure factors   

**Figure 7:**
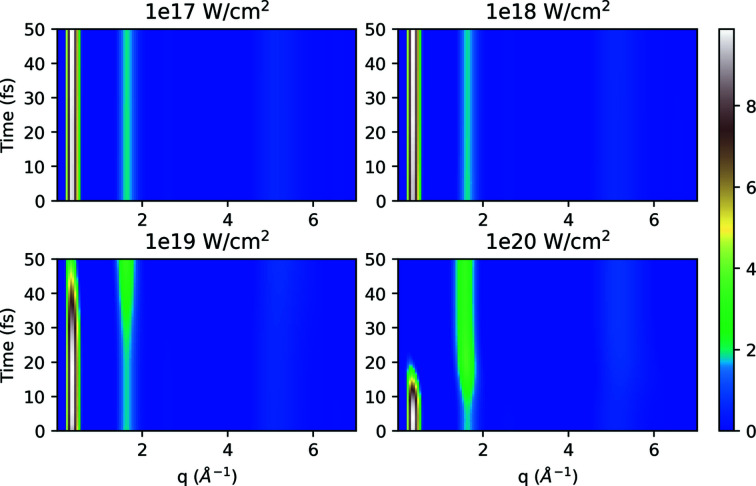
Time evolution of structure factor *S*(*q*, *t*) for the system A-cold at 5 keV photon energy for different intensities. The system A-cold is static in the MD simulations, and the time evolution here is driven through the changes in the form factors.

Our aim is to deconstruct the dynamics of the structure factor and to study intrinsically how it evolves during the pulse and in the aftermath of being exposed to the XFEL radiation. Following the procedure outlined earlier for the static case, we calculate the initial structure factor as a Fourier transform of the RDFs (*g*_αβ_), and in Fig. 7 ▸ (for system A-cold) we investigate the changes as a function of time and *q* as described in equation (7).

As expected, in Fig. 7 ▸ there are clearly distinct signal peaks visible in the spectrum. The peaks for the first two lowest intensities (1 × 10^17^, 1 × 10^18^ W cm^−2^) are almost constant, but as the intensities increase we see significant changes in the evolution depending on *q*, with the peak at low *q* decreasing in intensity as intermediate *q* peaks increase in intensity as pulse time progresses. This effect is driven by the time-dependent form factor which is in the denominator of the structure factor expression and it decreases at high intensities, ultimately resulting in an increase in *S*(*q*, *t*).

### 3.5. Effects of the atomic motion   

Time-dependent RDFs were calculated for the sample, where the system was simulated with XFEL parameters like photon energy, pulse intensity (1 × 10^19^ to 1 × 10^20^ W cm^−2^) and pulse duration (50 fs) in the classical MD box (system D-hot). We ran more than 100 independent simulations with different random seeds to obtain decent statistical accuracy. The atomic positions from different time frames were then extracted and combined together as a function of time for all the simulations. As a result, we created pulse-time-based structure files for the sample and then calculated RDFs of the elements as a function of time.

**Figure 8:**
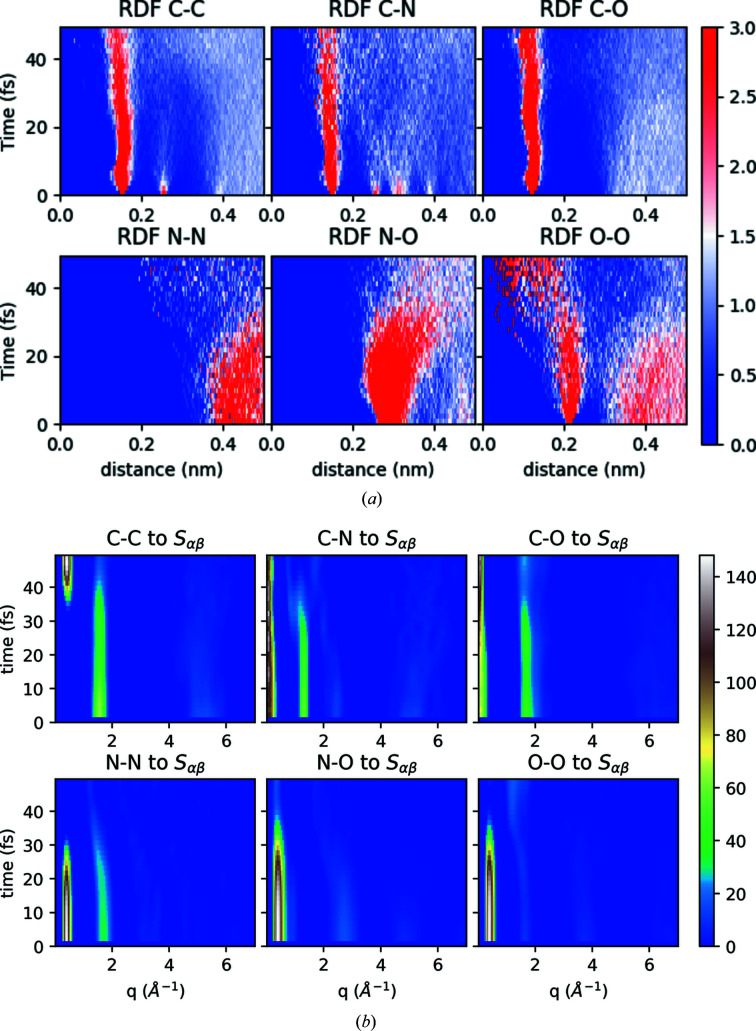
(*a*) Time evolution of RDFs for all pair-wise atoms for the system D-hot and (*b*) corresponding element-wise contribution to structure factor *S*_αβ_. The XFEL intensity for the MD simulations was 1 × 10^20^ W cm^−2^, matched with a corresponding intensity of 1 × 10^19^ W cm^−2^ for the plasma simulations, according to Fig. 2 ▸.

Figure 8 ▸(*a*) shows the time-dependent RDFs for different atom–atom distances. The plot indicates that the distance between the backbone atoms for the pentylammonium cation (like C–C, C–N) or formate anion (C–O) is still intact, but occupies a wider distribution of distances. However, the intermolecular distance between atoms like N–N, N–O or O–O is perturbed after around 30 fs and onwards, indicating the intermolecular meltdown of the system because of the XFEL radiation.

The element-wise contribution to the time-dependent structure factor from time-dependent atomic motions is shown in Fig. 8 ▸(*b*). The plot depicts how atomic motion plays an important role on the observed signal. As expected from previous considerations, here we see all the contributions to the peaks, at long range and short range. In particular, the N–N and N–O contribution at *q* values of around 2.5–3 Å^−1^ disappears on faster time scales (10 fs) than the contribution at 0.5 Å^−1^ (around 30 fs). The overall trend indicates that, for the initial time segments of the pulse duration, all the atomic bonds contribute with comparable strengths. As the time increases, the atomic displacement of the system becomes more evident in the scattering, since the bonds break and hence their corresponding contribution to the structure factor is lost, as seen in Fig. 8 ▸. The contribution from the backbone bonds, as we saw from the RDF plot, still holds up to around 30–40 fs while the other correlations start disappearing even earlier.

### 3.6. Total dynamics of the combined structure factor   

Finally, we are now able to construct the entire dynamics of the scattering, by combining the contributions from atomic motion with their changes in the RDF, and the ionization from the plasma with the subsequent changes in the form factor.

**Figure 9:**
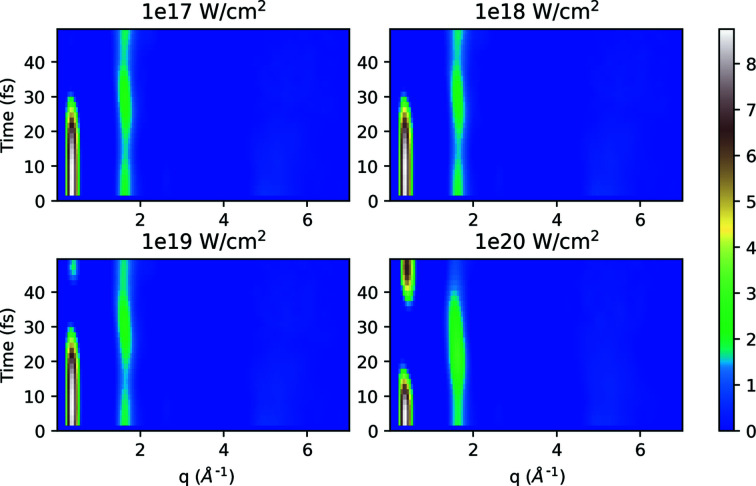
Time evolution of structure factor *S*(*q*, *t*) for the system D-hot at 5 keV photon energy with different intensities, including the time-dependent atomic displacement and time-dependent ionization.

Figure 9 ▸ represents the dynamics of the combined structure factor induced by the time-dependent ionization due to the X-ray beam conditions and atomic displacement of the sample (system D-hot), with the expression for *S*(*q*, *t*) as derived in equation (7). A comparison of the structure factor plot (Fig. 7 ▸), induced only by ionization of the sample to this latest plot that combines all effects, indicates that the loss of the signal is even more prominent here both at lower and higher *q* values. The signal at higher *q* (∼5 Å^−1^) is affected quite strongly and is smeared out for all our simulations at all photon energies and pulse intensities. For the peak corresponding to intermediate *q* (∼1.5–2 Å^−1^), the plot indicates that the changes are due to the impact of both the ionization and atomic displacement. Specifically in the first part of the pulse (0–20 fs) the ionization dominates and is the prime reason for the signal loss, and then at later stages (after 30 fs onwards) the atomic displacement of the sample comes into play and adds to the signal. The low *q* signal that corresponds to the long-range order is strongly affected by both ionization and atomic motion, and it even shows new long-range order appearing from the C–C between different cations [the contribution can be traced back to Fig. 8 ▸(*b*)].

Across the *q* range we see the combined effect of ionization and displacement affecting the scattering signal in different ways. The high resolution is smeared out due to the quick loss of coherence, as seen before in single-particle imaging and crystallography at XFELs. The intermediate *q* range shows a strong correlation of the C–C backbone (∼1.5–2 Å^−1^), but a fast disappearing of the signal from N–O at *q* ≃ 2.5 Å^−1^, which indicates that the correlation between the pentylammonium cation and the formate anion becomes fragile due to ionization. The signal in the low *q* range that is associated with the overall order of the ionic liquid (notably the N–N and N–O distances) persists for a duration of about 20–30 fs, but it is completely ‘terminated’ during the pulse due to the loss of scattering power of the ions. Thus, we find a strange inversion of suppression of scattering, where the low-resolution signal can disappear faster than intermediate resolutions, reflecting the properties of the liquid and X-ray scattering.

Figures 10–15 of the supporting information show the entire set of results, with four choices of X-ray beam intensity and four choices of photon energies, for both systems A-cold and D-hot, as well as the form factors for the three elements: carbon, nitrogen and oxygen.

## 4. Summary and outlook   

In this paper we give a detailed presentation of our methodology for combining classical molecular dynamics (*GROMACS*) and non-thermal plasma simulations (*CRETIN*) to investigate the ultrafast phase transition of a protic ionic liquid (pentylammonium formate) to plasma, initiated and probed by an XFEL pulse. A typical experiment at an XFEL will use femtosecond X-ray pulses to record scattering from liquid samples, and, while the pulse propagates through the sample and quickly turns it into a plasma, the recorded diffraction pattern provides an average picture of the entire dynamics. We use the combination of software packages to unravel the different contributions to the dynamics, with the possibility of explicitly turning on/off some interactions and propagate their effects towards the final result.

We performed *GROMACS* simulations on several sets of simulation boxes with different conditions, and calculated the atom dynamics, the RDFs and the scattering factors, that indicate how different atom pairs contribute to the final result. We find that different atomic pairs have dynamics that disappear faster during the pulse, for example the N–O and N–N distances associated with long order in the liquid, while others are more robust, for example the C–C distances in the backbone of the cations. We simulated with *CRETIN* for a range of XFEL starting parameters (photon energy, beam intensity, with a fixed pulse duration), and calculated the ionization dynamics and the atomic and ionic form factors that were also traced in the final results. We see how the loss of electrons has a higher impact in the scattering especially toward the later part of the pulse (30 fs and onwards), and also as a function of resolution, with features at higher resolution being smeared out faster and more drastic than lower resolutions. These findings are in line with previous studies on diffraction from protein crystals and single particles, and paint a rather complex, but manageable, picture of the effect of ionization and ion motion in a liquid during the pulse as a function of the resolution.

These studies are relevant for future experiments aimed at investigating the structure of protic ionic liquids or other organic liquids with XFEL pulses, which could also be used to benchmark our results. Ultrafast XFEL pulses can be used to outrun the diffusion time in liquids, and there have been suggestions to use XFELs to study the local 3D structure of a liquid. The key challenges are the development of data collection and analysis methods to extract the signal (Martin, 2017 ▸). Protic ionic liquids are suitable for such experiments because they exhibit nanostructure beyond 1–2 nm length scales and provide a strong 3D structural signal. They are also being explored as non-aqueous bio-solvents for proteins and protein crystallization (Han *et al.*, 2021 ▸), and may have favourable properties for sample delivery in XFEL experiments. Furthermore, the dynamics on the femtosecond time scale can be unravelled experimentally using XFEL-pump/XFEL-probe schemes, which are increasingly available modes of operation at XFELs around the world.

## Supplementary Material

Supporting resulting figures: Figures 10 to 15; Example input file for GROMACS; Example input file for CRETIN. DOI: 10.1107/S1600577521007657/xh5057sup1.pdf

## Figures and Tables

**Table 1:** Parallel MD simulation models built with different parameters starting from the basic system A-cold to the system D-hot The RDFs calculated from each of the systems were used separately with ionization form factors from plasma results to calculate the total structure factor of the system. The XFEL parameters show the intensity, pulse duration and photon energy.

System	Temperature (K)	Ionization	XFEL parameters	RDF
A-cold	298	No	No	Static
B	10000	No	No	Static
C	10000	Yes	1 × 10^20^ W cm^−2^, 50 fs, 8 keV	Static
D-hot	10000	Yes	1 × 10^20^ W cm^−2^, 50 fs, 8 keV	Dynamic

## Footnotes

1 For a comparison of XFEL sources and parameters, see https://www.xfel.eu/facility/comparison/.
