# Supplementary material for: Ultrafast dynamics and scattering of protic ionic liquids induced by XFEL pulses
Source: J Synchrotron Radiat. 2021 Aug 19;28(Pt 5):1296–308. doi: 10.1107/S1600577521007657 (PMC8415341; doi:10.1107/S1600577521007657)
Supplement: Supplementary file 1 [file s-28-01296-sup1.pdf]

JOURNAL OF SYNCHROTRON RADIATION

Volume 28 (2021)

Supporting information for article:

Ultrafast dynamics and scattering of protic ionic liquids induced by XFEL pulses

Kajwal Kumar Patra, Ibrahim Eliah Dawod, Andrew V. Martin, Tamar L. Greaves, Daniel Persson, Carl Caleman and Nicusor Timneanu

Appendix A
Supplementary figures

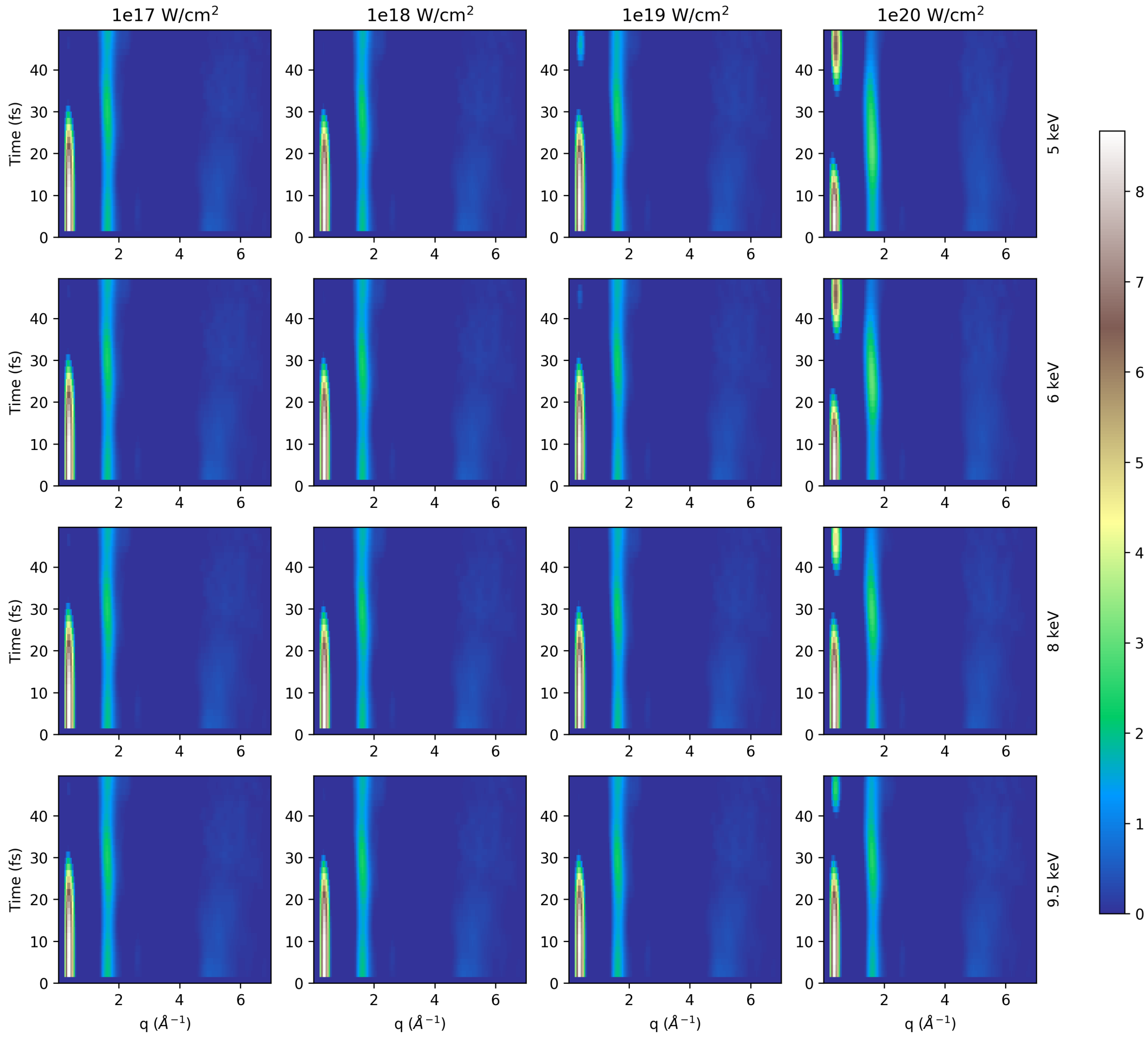

Fig. 10. Time evolution of structure factor $S(q, t)$ for the system D-*hot* at 5 to 9.5 keV photon energy with different intensities (1e17 to 1e20 W/cm^2).

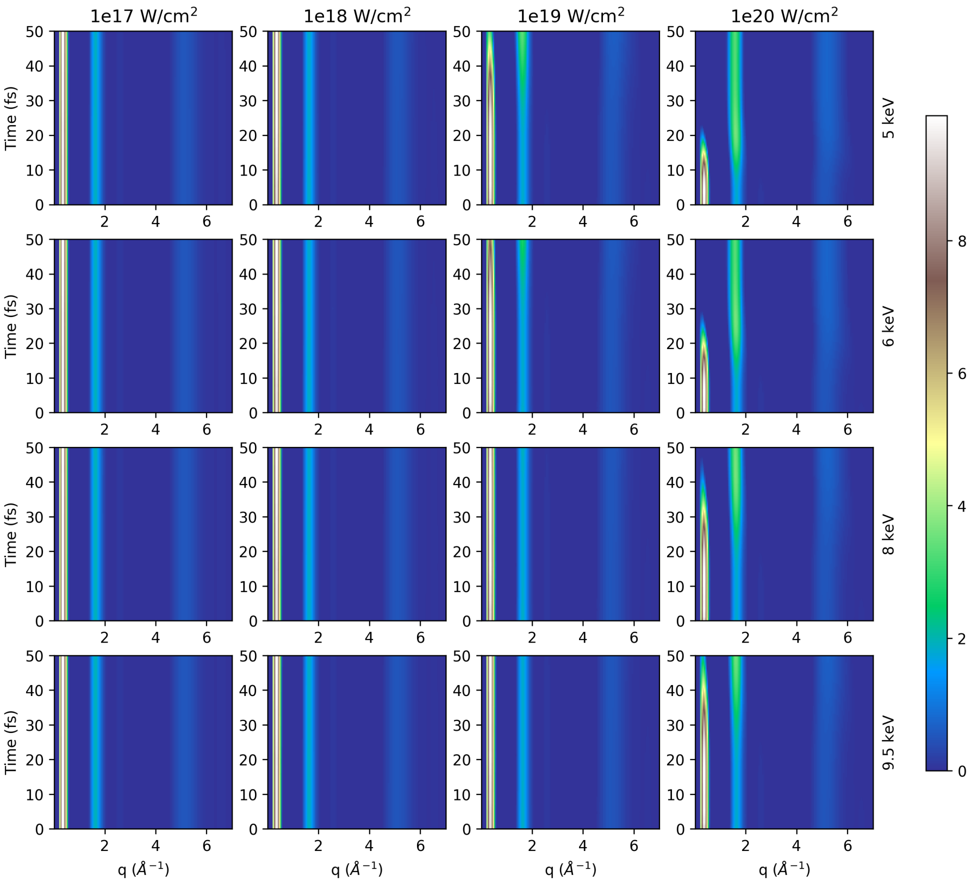

Fig. 11. Time evolution of structure factor $S(q,t)$ for the system A-*cold* at 5 to 9.5 keV photon energy with different intensities (1e17 to 1e20 W/cm^2).

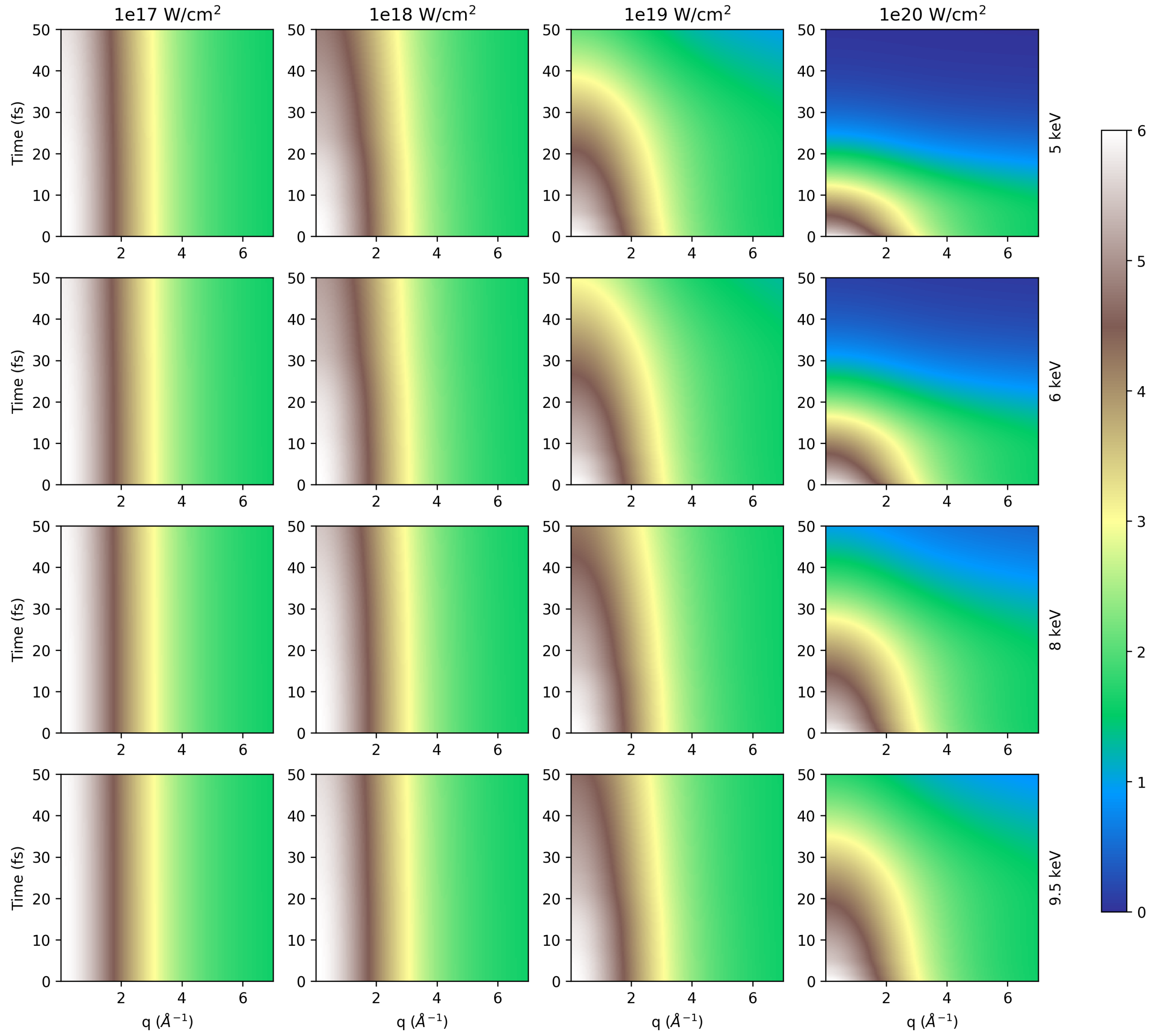

Fig. 12. Time evolution of carbon factor factors following plasma simulations at 5 to 9.5 keV photon energy with different pulse intensities (1e17 to 1e20 W/cm^2).

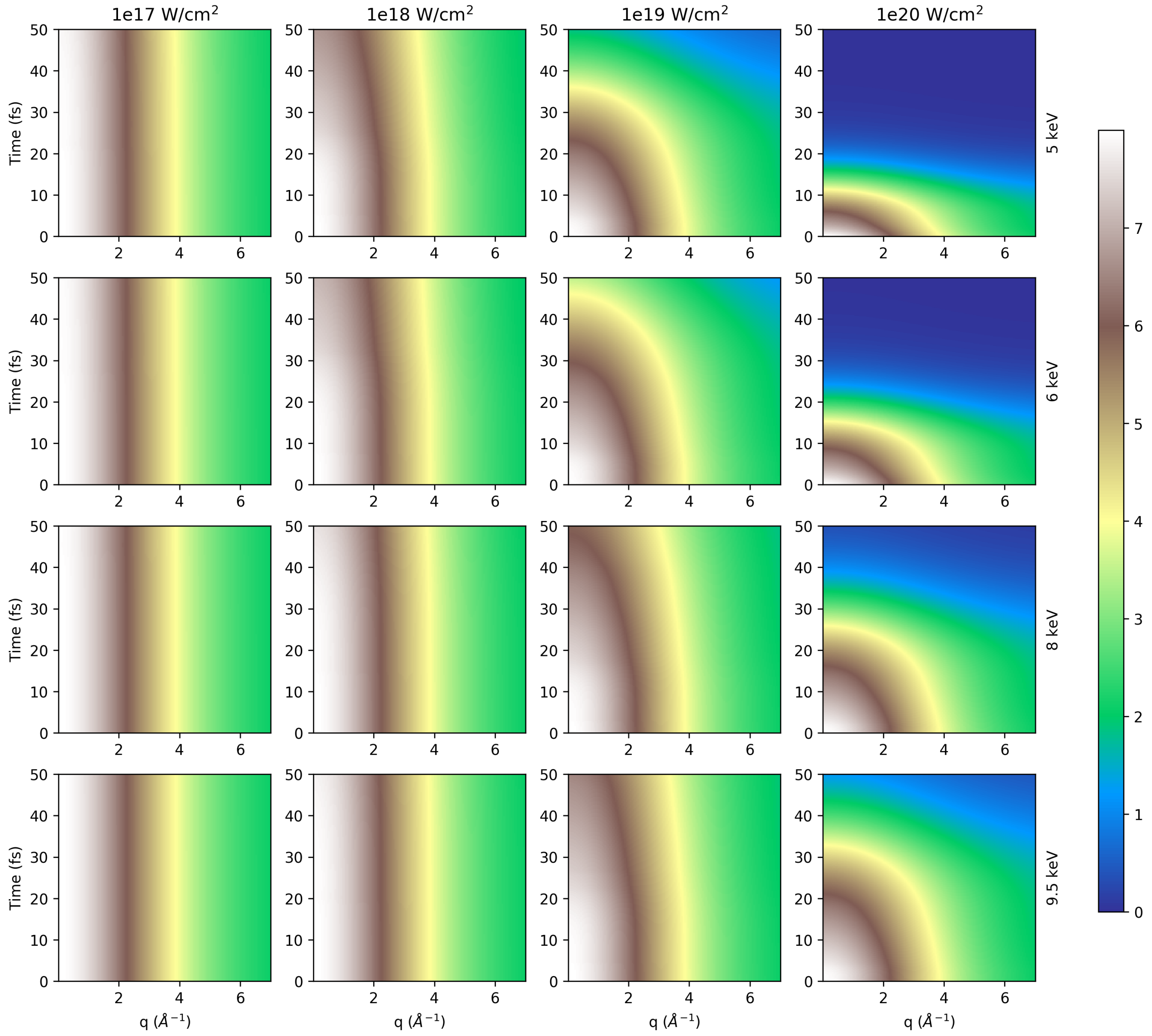

Fig. 13. Time evolution of nitrogen factor factors following plasma simulations at 5 to 9.5 keV photon energy with different pulse intensities (1e17 to 1e20 W/cm^2).

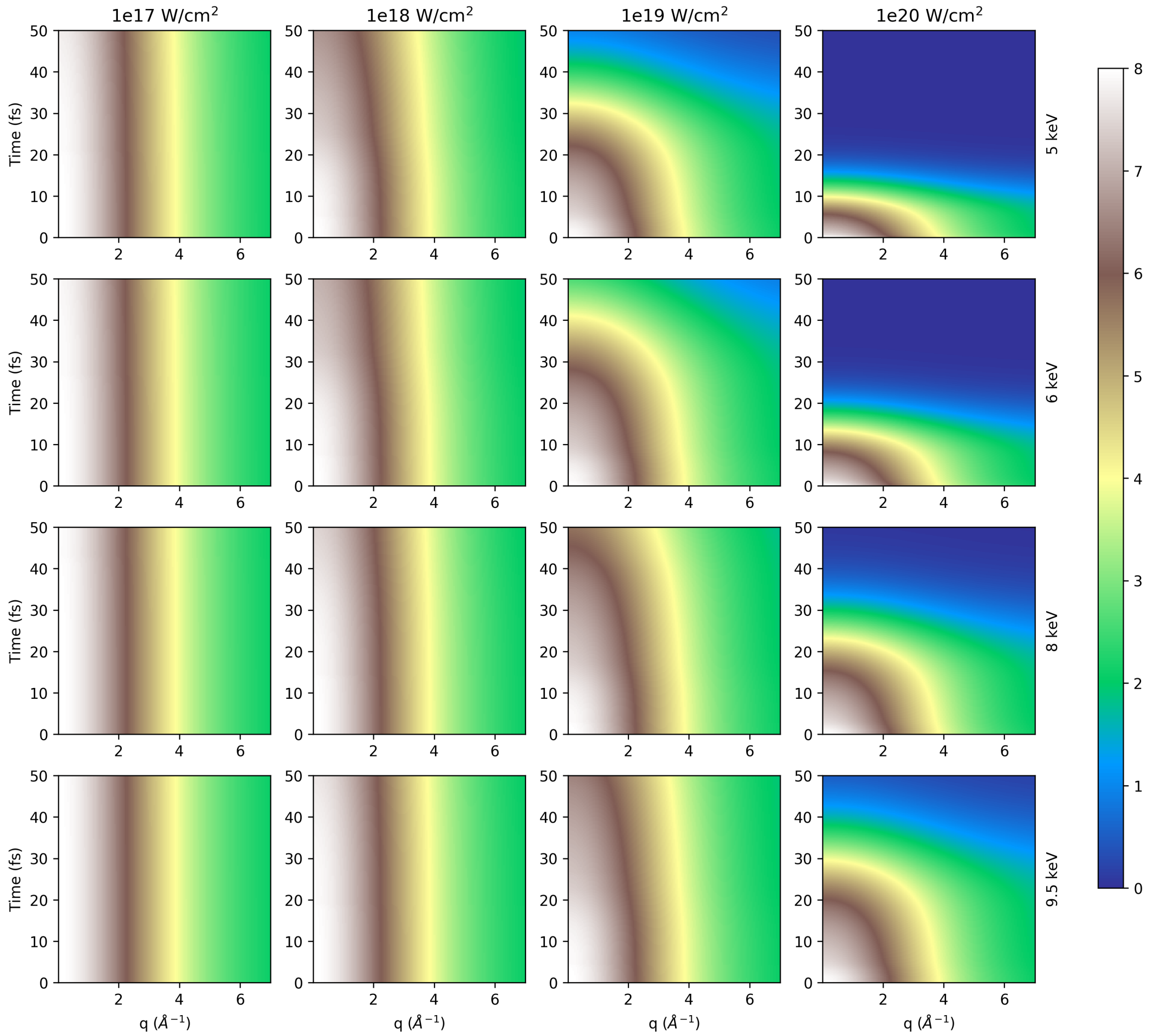

Fig. 14. Time evolution of oxygen factor factors following plasma simulations at 5 to 9.5 keV photon energy with different pulse intensities (1e17 to 1e20 W/cm^2).

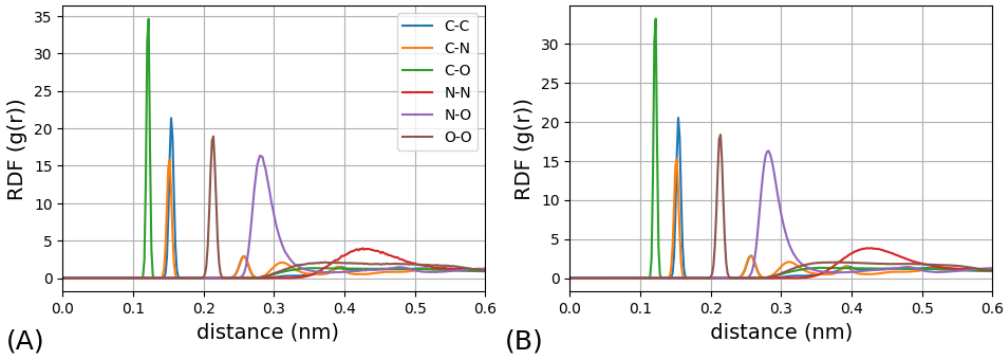

Fig. 15. The static RDF of element pairs present in the PeAF, calculated from the classical MD simulation trajectory of the A-*cold* system for two different box sizes: (A) a small box with 300 PeAF ion pairs and (B) a large box with 1200 PeAF ion pairs. The RDFs and structure factors $S(q)$ are nearly identical within the statistical accuracy of the simulation.

Appendix B
Example of a GROMACS input

GROMACS is a free software available under the GNU Lesser General Public License. The equilibration simulations were performed with the 2019 version, which is available from the website https://manual.gromacs.org/ . The explosion simulations were performed with the version 3.3.3 (legacy code) and the source code can be obtained from the website https://www.gromacs.org/ . Newer versions of GROMACS have removed the explosion simulations in favour of optimising the speed of simulations with rewritten algorithms (including CPU and GPU parallelisation), which was incompatible with the original calculation of the Coulomb explosions. Contact the authors for support in running the legacy code. Below is an example of an input file (Example_gromacs.mdp) containing the starting parameters of the explosion simulations.

Appendix C

Example of a CRETIN input

CRETIN is a licensed software developed at the Lawrence Livermore National Laboratory. The software package can be obtained through a collaboration agreement with the LLNL Inovation and Partnership Office. For these simulations we use the version 2.13. Below is an example of a generator file (Example_cretin.gen) with the parameters and switches of the simulation.

```
; VARIOUS PREPROCESSING OPTIONS
title                    = PeAF
cpp                      = /lib/cpp

; RUN CONTROL PARAMETERS
integrator               = md
; start time and timestep in ps
tinit                    = 0
dt                       = 1e-05
; number of steps
nsteps                   = 10000
; mode for center of mass motion removal
comm-mode                = Linear
; number of steps for center of mass motion removal
nstcomm                  = 1

; LANGEVIN DYNAMICS OPTIONS
; Temperature, friction coefficient (amu/ps) and random seed
bd-temp                  = 300
bd-fric                  = 0
ld-seed                  = 2006

; ENERGY MINIMIZATION OPTIONS
; Force tolerance and initial step-size
emtol                    = 100
emstep                   = 0.01
; Max number of iterations in relax_shells
niter                    = 20
; Step size (1/ps^2) for minimization of flexible constraints
fcstep                   = 0
; Frequency of steepest descents steps when doing CG
nstcgsteep               = 1000

; OUTPUT CONTROL OPTIONS
; Output frequency for coords (x), velocities (v) and forces (f)
nstxout                  = 5
nstvout                  = 5
nstfout                  = 5
; Output frequency for energies to log file and energy file
nstlog                   = 1
nstenergy                = 1
; Output frequency and precision for xtc file
nstxtcout                = 0
xtc-precision            = 1000

; NEIGHBORSEARCHING PARAMETERS
; nblist update frequency
nstlist                  = 5
; ns algorithm (simple or grid)
ns_type                  = simple
; Periodic boundary conditions: xyz or no
pbc                      = xyz
; nblist cut-off
```

```
rlist                    = 0.6
domain-decomposition     = no

; OPTIONS FOR ELECTROSTATICS AND VDW
; Method for doing electrostatics
coulombtype              = pme
rcoulomb-switch          = 0
rcoulomb                 = 0.6
; Dielectric constant (DC) for cut-off or DC of reaction field
epsilon-r                = 1
; Method for doing Van der Waals
vdw-type                 = Cut-off
; cut-off lengths
rvdw-switch              = 0
rvdw                     = 0.6
; Apply long range dispersion corrections for Energy and Pressure
DispCorr                 = EnerPres
; Spacing for the PME/PPPM FFT grid
fourierspacing           = 0.10
; FFT grid size, when a value is 0 fourierspacing will be used
fourier_nx               = 0
fourier_ny               = 0
fourier_nz               = 0
; EWALD/PME/PPPM parameters
pme_order                = 4
ewald_rtol               = 1e-05
ewald_geometry           = 3d
epsilon_surface          = 0
optimize_fft             = no

; OPTIONS FOR WEAK COUPLING ALGORITHMS
; Temperature coupling
Tcoupl                   = no
; Groups to couple separately
tc-grps                  = System
; Time constant (ps) and reference temperature (K)
tau_t                    = 0.00001
ref_t                    = 10000
; Pressure coupling
Pcoupl                   = no
Pcoupltype               = isotropic
; Time constant (ps), compressibility (1/bar) and reference P (bar)
=
tau_p                    = 0.5
compressibility          = 4.5e-5
ref_p                    = 1.0

; SIMULATED ANNEALING CONTROL
annealing                = no
; Time at which temperature should be zero (ps) =
zero-temp_time           = 0

; GENERATE VELOCITIES FOR STARTUP RUN
gen_vel                  = yes
```

```
gen_temp                 = 10000
gen_seed                 = 100

; OPTIONS FOR BONDS
constraints              = none
; Type of constraint algorithm
constraint-algorithm     = Lincs
; Do not constrain the start configuration
unconstrained-start      = no
; Use successive overrelaxation to reduce the number of shake
iterations =
Shake-SOR                = no
; Relative tolerance of shake
shake-tol                = 1e-04
; Highest order in the expansion of the constraint coupling matrix
lincs-order              = 4
; Lincs will write a warning to the stderr if in one step a bond
; rotates over more degrees than
lincs-warnangle          = 30
; Convert harmonic bonds to morse potentials
morse                    = yes

; NMR refinement stuff
; Distance restraints type: No, Simple or Ensemble
disre                    = No
; Force weighting of pairs in one distance restraint: Conservative
or Equal
disre-weighting          = Conservative
; Use sqrt of the time averaged times the instantaneous violation
disre-mixed              = no
disre-fc                 = 1000
disre-tau                = 0
; Output frequency for pair distances to energy file
nstdisreout              = 100
; Orientation restraints: No or Yes
orire                    = no
; Orientation restraints force constant and tau for time averaging
orire-fc                 = 0
orire-tau                = 0
; Output frequency for trace(SD) to energy file
nstorireout              = 100

; Free energy control stuff
free-energy              = no
init-lambda              = 0
delta-lambda             = 0
sc-alpha                 = 0
sc-sigma                 = 0.3

; USER DEFINED PULSE PARAMETRES
;random seed for stoch ionization
userint1                 = 611
;photon energy keV
userint2                 = 8
```

```
;peak of the pulse, in time
userreal1                = 0.0
;intensity in photon/pulse
userreal2                = 1.0e14
;pulse length (50 fs) sigma
userreal3                = 0.01274
;spotsize diameter nm > intensity per micron2
userreal4                = 1000
```

```
c   *** 1D Hydro-Kinetics for PeAF in XFEL ***

c -------------------------------------------------------------
c   Aliases
c -------------------------------------------------------------
c NUMBER OF ZONES IN 1D
alias N        6

c INITIAL ELECTRON DENSITY, SHOULD BE LESS THAN MELTING
c THRESHOLD FOR ABLATION SIMULATIONS.
alias TE       0.01

c DENSITY OF SAMPLE in gm/cm3
alias RHO  0.93

c SPECTRAL WIDTH OF LASER RADIATION IN eV
alias E1        8000
alias E2        E1 * 1.0104

c REGIONS IN 1D, E.G VACUUM AND SOLID DELIMITED BY
c n1-n3
alias n0 1
alias n1 N

c CHEMICAL STOICHIOMETRY
c 133.187 id the molecular weight of sample PeAF (C6+N1+O2+H15)
alias N_o  2. * RHO * AVGD / 133.187
alias N_h 15. * RHO * AVGD / 133.187
alias N_c  6. * RHO * AVGD / 133.187
alias N_n  1. * RHO * AVGD / 133.187


c LASER INTENSITY [erg/cm2/s]
alias ILASER 1.000e+27

c -------------------------------------------------------------
c   Materials
c -------------------------------------------------------------

c DECLARE THAT THE INTRINSIC HYDOGRNIC MODEL
c SHOULD BE USED FOR ALL ELEMENTS
c outer
atoms hydrogenic o
atoms hydrogenic h
atoms hydrogenic c
atoms hydrogenic n

c DEFINING THE MATERIAL REGIONS
region  n0 n1  TE
 element 1 N_o
 element 2 N_h
 element 3 N_c
 element 4 N_n
```

```
c ------------------------------------------------------------
c   Geometry
c ------------------------------------------------------------

c LINEAR SPACING FOR THE ZONES
 geometry plane

rlin n0 n1 0. 5e-4

c ------------------------------------------------------------
c   Radiation
c ------------------------------------------------------------

angles 1

c SPECTRAL BINS, CAN BE REFINED CLOSE TO EDGES AND
c STRONG LINES
 ebins 60 0.01 30.0
 ebins 60  30.0 400.0
 ebins 60  400.0 5.e4

c ------------------------------------------------------------
c   Sources
c ------------------------------------------------------------

alias MULT HPEV / ( E2 - E1)

c INCOMING LASER
 source jbndry 1 E1 E2 value history 1 MULT
 xfilebc 1  1. 0. 1. 1                  ! normal incidence, isotropy

c GAUSSIAN LASER PULSE, 100.e-15 IS THE "TIME UNIT"
 history 1 ILASER 1.e-15 gaussian 50 30

c ------------------------------------------------------------
c   Controls
c ------------------------------------------------------------
c START AND END TIME OF SIMULATION
tstart 0.
tquit  1.000e-13

c ------------------------------------------------------------
c   Switches and Parameters
c ------------------------------------------------------------

switch 11 2                   ! make .plt file
switch 20 1                   ! NLTE
switch 25 1                   ! time-dependent

switch 28 0                   ! LTE initialization
switch 31 1                   ! temperature calculation
switch 44 250                 ! maximum # of iterations
c switch 52 -1                  ! Stark broaden everything
```

```
switch 72  1                   ! timesteps between spectral
calculations
switch 127 2
switch 126 10
switch 29 1
c switch 12 -1

c ------------- HYDRO STUFF ----------------

switch 2 1

switch 125 1                   ! average spectral emission over bins
switch 55  1                       ! continuum lowering
switch 17  1                     ! include line contributions in
opacity
switch 36 1                     ! turn on continuum transfer
switch 133 10002
switch 35 -1
switch 155 1
switch 146 1                   ! turn on multiphoton ionization


c param 23 1.0
param 40 1.e-16                ! time between edits
param 41 1.e-17                ! initial timestep
c param 44 1.e-17
param 45 2.e-16                ! maximum timestep
param 61 1.e-3                 ! iso-sequence population threshold
param 95 0.01                 ! minimum Zbar for kinetics
param 102 0.01               ! minimum temperature
c param  96 0.7

c ------------------------------------------------------------
c   Edits
c ------------------------------------------------------------
c OUTPUT PARAMETERS TO BE PRINTED IN THE OUTPUT(.plt) FILE
plot "depth vs zbar"
xvar r
yvar ne
yvar zbar 1:4
yvar tev
yvar tiv
yvar cspd
yvar pmat
yvar tauii
yvar vel
yvar ytot 1
yvar ytot 4
yvar ytot 3

plot "t vs oxygen"
xvar r
yvar yisofrac 1 0 8
yvar yisofrac 1 0 7
```

```
yvar yisofrac 1 0 6
yvar yisofrac 1 0 5
yvar yisofrac 1 0 4
yvar yisofrac 1 0 3
yvar yisofrac 1 0 2
yvar yisofrac 1 0 1
yvar yisofrac 1 0 0

plot "t vs nitrogen"
xvar r
yvar yisofrac 4 0 7
yvar yisofrac 4 0 6
yvar yisofrac 4 0 5
yvar yisofrac 4 0 4
yvar yisofrac 4 0 3
yvar yisofrac 4 0 2
yvar yisofrac 4 0 1
yvar yisofrac 4 0 0

plot "t vs carbon"
xvar r
yvar yisofrac 3 0 6
yvar yisofrac 3 0 5
yvar yisofrac 3 0 4
yvar yisofrac 3 0 3
yvar yisofrac 3 0 2
yvar yisofrac 3 0 1
yvar yisofrac 3 0 0

c ----------- Oxygen States -----------------------

plot "r vs 0 K-shell electrons Oxygen"
xvar r
yvar yn_0 1 0 0:8

plot "r vs fraction 0 K-shell electrons Oxygen"
xvar r
yvar ynf_0 1 0 0:8

plot "r vs 1 K-shell electrons Oxygen"
xvar r
yvar yn_1 1 0 0:8

plot "r vs fraction 1 K-shell electrons Oxygen"
xvar r
yvar ynf_1 1 0 0:8

plot "r vs 2 K-shell electrons Oxygen"
xvar r
yvar yn_2 1 0 0:8

plot "r vs fraction 2 K-shell electrons Oxygen"
xvar r
yvar ynf_2 1 0 0:8
```

```
c ----------- Nitrogen States -----------------------

plot "r vs 0 K-shell electrons Nitrogen"
xvar r
yvar yn_0 4 0 0:7

plot "r vs fraction 0 K-shell electrons Nitrogen"
xvar r
yvar ynf_0 4 0 0:7

plot "r vs 1 K-shell electrons Nitrogen"
xvar r
yvar yn_1 4 0 0:7

plot "r vs fraction 1 K-shell electrons Nitrogen"
xvar r
yvar ynf_1 4 0 0:7

plot "r vs 2 K-shell electrons Nitrogen"
xvar r
yvar yn_2 4 0 0:7

plot "r vs fraction 2 K-shell electrons Nitrogen"
xvar r
yvar ynf_2 4 0 0:7

c ----------- Carbon States -----------------------

plot "r vs 0 K-shell electrons Carbon"
xvar r
yvar yn_0 3 0 0:6

plot "r vs fraction 0 K-shell electrons Carbon"
xvar r
yvar ynf_0 3 0 0:6

plot "r vs 1 K-shell electrons Carbon"
xvar r
yvar yn_1 3 0 0:6

plot "r vs fraction 1 K-shell electrons Carbon"
xvar r
yvar ynf_1 3 0 0:6

plot "r vs 2 K-shell electrons Carbon"
xvar r
yvar yn_2 3 0 0:6

plot "r vs fraction 2 K-shell electrons Carbon"
xvar r
yvar ynf_2 3 0 0:6
```
